# Application of microarray technology for the detection of intracranial bacterial infection

**DOI:** 10.3892/etm.2013.1443

**Published:** 2013-12-10

**Authors:** JIANHONG SHEN, YIXIANG GUAN, JIANPING ZHANG, JIANWU TANG, XIAOJIAN LU, CHUNXIU ZHANG

**Affiliations:** 1 Department of Neurosurgery, Affiliated Hospital of Nantong University, Nantong, Jiangsu 226001, P.R. China; 2 Department of Surgery, Affiliated Haian People’s Hospital of Nantong University, Haian, Jiangsu 226600, P.R. China; 3 Department of Infectious Disease, Affiliated Haian People’s Hospital of Nantong University, Haian, Jiangsu 226600, P.R. China; 4 Shanghai Biochip National Engineering Research Center, Shanghai 201203, P.R. China

**Keywords:** intracranial infection, cerebrospinal fluid, bacterial culture, microarray, drug resistance gene

## Abstract

The aim of this study was to assess the value of microarray technology for the detection of intracranial bacterial infection. A small gene chip was prepared based on the four pathogens commonly known to cause intracranial infection and the corresponding six types of common resistance genes in The Affiliated Hospital of Nantong University and The Affiliated Haian People’s Hospital of Nantong University. Cerebrospinal fluid samples were then collected from 30 patients with clinically diagnosed intracranial infection for the detection of the bacteria and resistance genes. The results were compared with the bacterial culture and sensitivity test results from the Department of Clinical Laboratories. The laboratory bacterial culture took 4–5 days, and revealed that 12 cases were positive and 18 cases were negative for bacteria. The microarray analysis took 1 day, and bacteria and resistance genes were detected in 15 cases. The 16S gene and drug resistance genes were detected in 8 cases; however, the bacterial strain was not identified. Seven cases appeared negative for bacteria and resistance genes. Microarray technology is rapid, sensitive and suitable for use in the detection of intracranial infections and other diseases for which conventional bacterial culture has a low positive rate.

## Introduction

Bacterial infection of the central nervous system is a common and serious threat to human life, and requires timely and effective antibiotic treatment (1). For this to occur, a rapid and accurate detection and identification of the cerebrospinal fluid (CSF) pathogens is necessary. The diagnosis of intracranial infection mainly relies on CSF bacterial culture, which is considered to be the gold standard due to its high specificity (2,3). However, this is a time consuming procedure, and specific bacteria are difficult to cultivate. In addition, given the influence of antibiotics, among other factors, the positive rate of CSF culture is minimal, i.e. ~10% in the hospital and 10–20% in the majority of studies (3,4). Therefore, the current methods of etiological examination are inadequate when compared with the advancements in clinical treatment (1). Microarray technology has the advantages of easy operation, rapid detection and the ability to simultaneously detect a large number of specific molecules. In the present study, several types of typical intracranial infection-causing pathogens and their common resistance genes were selected based on their specific DNA sequence. A small microarray was designed and prepared to study the application value of microarray technology in intracranial infection.

## Materials and methods

### Types of bacteria and drug resistance genes

Based on the common intracranial infection-causing bacteria indicated by the Neurosurgery Department of The Affiliated Hospital of Nantong University (Nantong, China) and The Affiliated Haian People’s Hospital of Nantong University (Haian, China), the following Gram-positive and Gram-negative cocci and bacilli were used: *Staphylococcus aureus, Klebsiella pneumoniae, Escherichia coli* and *Streptococcus pneumoniae*. In addition, the six most common drug resistance genes of these four bacteria (mecA, OXA-23, SHV, CTX-M, TEM and PBP1a) were tested. This study was conducted in accordance with the Declaration of Helsinki and with approval from the Ethics Committee of the Affiliated Haian People’s Hospital of Nantong University (Haian, China). Written informed consent was obtained from all participants.

### Clinical specimen collection

Based on the detected stains, 30 CSF samples from patients with clinically diagnosed intracranial infection were collected between January 2010 and August 2011 at the Neurosurgery Departments of the Affiliated Hospital of Nantong University (Nantong, China) and the Affiliated Haian People’s Hospital of Nantong University. Among the samples, bacterial culture revealed that 12 cases were positive (five for *S. aureus*, three for *K. pneumoniae*, two for *E. coli* and two for *S. pneumoniae*) and 18 cases were negative (the negative CSF samples within the same period were generated numbers and randomly selected). The clinical diagnosis of intracranial bacterial infection was based on the Harrison standard (5), combined with the following neurosurgical characteristics: i) Risk factors for intracranial infection, including CSF leak, open brain injury, surgery for an extended period (>4 h), >2 surgeries and external drainage of CSF; ii) clinical manifestations of fever, headache, vomiting or meningeal irritation; and iii) white blood cell count >1.18×10^9^cells/l, glucose <1.9 mmol/l and protein >2.2g/l, as assessed with CSF testing.

### CSF bacterial culture

Loop-picked turbid CSF was inoculated on blood agar and chocolate agar plates at 35°C under 5% CO_2_ for 24 h.

### Bacterial DNA extraction from CSF

Each CSF sample (~2 ml) was collected and centrifuged at 8,000 × g for 10 min. The supernatant was then discarded and the sediment was suspended in sterile saline, prior to being subjected to centrifugation at 8,000 × g for 10 min. Having discarded the supernatant, a DNeasy^®^ Blood & Tissue kit (Qiagen GmbH, Hilden, Germany) was used. DNA lysate (~180 μl) was added to the sediment, the mixture was placed in a 37°C water bath for 30 min and ~25 μl proteinase K being added. The mixture was subsequently placed in a 56°C water bath for 30 min and 200 μl ethanol was added using spin columns to extract the sample DNA.

### Primer design and synthesis of probes

The specific DNA sequences were screened for four types of bacteria and six resistance genes from GenBank (http://www.ncbi.nlm.nih.gov/genbank/), using the software Primer Premier 5.0 (Premier Biosoft, Palo Alto, CA, USA) to design 10 pairs of PCR primers and probes (Table I). The 16S rDNA gene codes for prokaryotic ribosomal small subunit rRNA (16S rRNA) and is the most common and useful ‘molecular clock’ in bacterial taxonomic studies. The constant region of 16S rRNA is a common feature of all bacteria (6). Therefore, based on the constant region of the 16S gene found in all strains of bacteria, primers and a probe were designed as a positive reference. All primers and probes were synthesized by Shanghai Invitrogen Biotechnology Co., Ltd. (Shanghai, China).

### Multiplex polymerase chain reaction (PCR)

The experiments were divided into two groups (Table I). The first group underwent microbiological testing, while the second group underwent resistance gene testing with water as a negative control. The reaction system contained the following: 1.5 μl buffer (10X), 0.2 μl deoxyribonucleotide triphosphate (10 mmol/l), 1.0 μl DNA (20 ng/μl), 0.2 μl *Taq* enzyme, 0.2 μl primers ×2 (20 μmol/l), 0.6 μl MgCl_2_ (25 mmol/l) and 11.1 μl H_2_O. The total volume was 15 μl (T_m_, 56°C; 30 cycles). The PCR products were analyzed using agarose gel electrophoresis (2% agarose; voltage, 150 V; running time, 15 min), and the bands were observed.

### Microarray preparation

**Figure 1:**
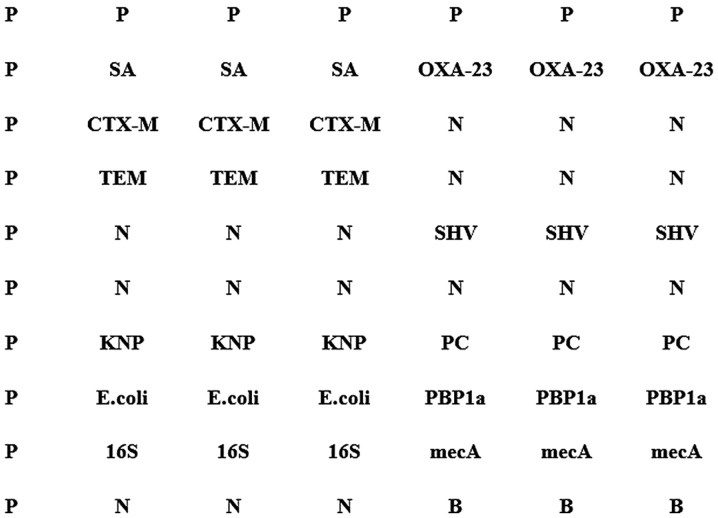
Arrangement picture of the bacteria and resistance gene-detecting microarray. N, negative control probe; P, positioning probe; B, blank control; SA, *Staphylococcus aureus*; KNP, *Klebsiella pneumoniae*; PC, *Streptococcus pneumoniae; E. coli*, *Escherichia coli*.

The PCR products were diluted to 50 mmol/l with spotting solution and added to 384-well plates (Corning Life Sciences, Tewksbury, MA, USA) at 10 μl per well. Under 60% humidity and 25°C, contact spotting (Omni Grid™ 100 microarray spotter; GeneMachine, USA) was performed to load the probe point to the optical level of the aldehyde modification chip (Boao Biology Co., Ltd., Beijing, China). The matrix measured 10×7, and all points were randomly arranged. Each probe set was repeated in triplicate (Fig. 1). The microarray was supplied by Shanghai Biochip Co., Ltd. (Shanghai, China) and placed in the oven during storage.

### Microarray hybridization and result interpretation

The PCR products were fluorescently labeled under the following conditions: 96°C for 3 min, followed by 66°C for 30 sec, 72°C for 20 sec for 35 cycles and extension at 72°C for 5 min. The products were then placed in the dark at 4°C. The fluorescently-labeled products were subjected to DNA hybridization (Thermo Hybaid Maxi 14 Hybridization Oven; Thermo Hybaid, Ulm, Germany) at 48°C for 2 h. The hybridized chip was then scanned (GenePix^®^ 4000B confocal laser scanner; Molecular Devices, LLC, Sunnyvale, CA, USA), and GenePix^®^ Pro 6.0 Acquisition and Analysis Microarray Software (Molecular Devices, LLC) was used to assess the fluorescence signal intensity value of each probe set. The low-signal locus was removed, and values higher than the cutoff value [signal-to-noise ratio, 3.0] were deemed as a valid signal.

## Results

### Multiplex PCR

**Figure 2:**
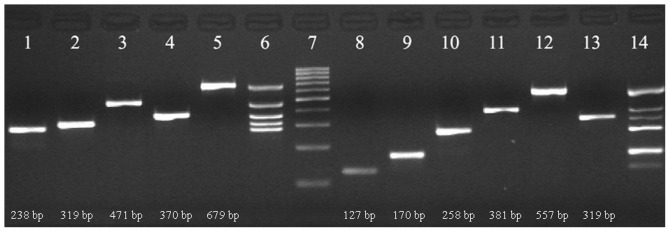
Detection of bacterial strains and resistance genes using electrophoresis of the PCR products. Lane 1, *Staphylococcus aureus*; 2, *Klebsiella pneumoniae*; 3, *Escherichia coli*; 4, 16S; 5, *Streptococcus pneumoniae*; 6, multiplex PCR of bacteria; 7, Marker; 8, OXA-23; 9, mecA; 10, TEM; 11, CTX-M; 12, PBP1a; 13, SHV; 14, multiplex PCR of resistance genes. The marker strips from the bottom to the top were: 100, 200, 300, 400, 500, 600, 700, 800, 900 and 1,000 bp. PCR, polymerase chain reaction.

Ten pairs of primers were designed to amplify the corresponding target bacteria and resistance gene sequences. The PCR products were then subjected to agarose gel electrophoresis, showing clear bands of the appropriate size (Fig. 2). The results demonstrated the specification and effectiveness of the designed primer sequences.

### Microarray hybridization

**Figure 3:**
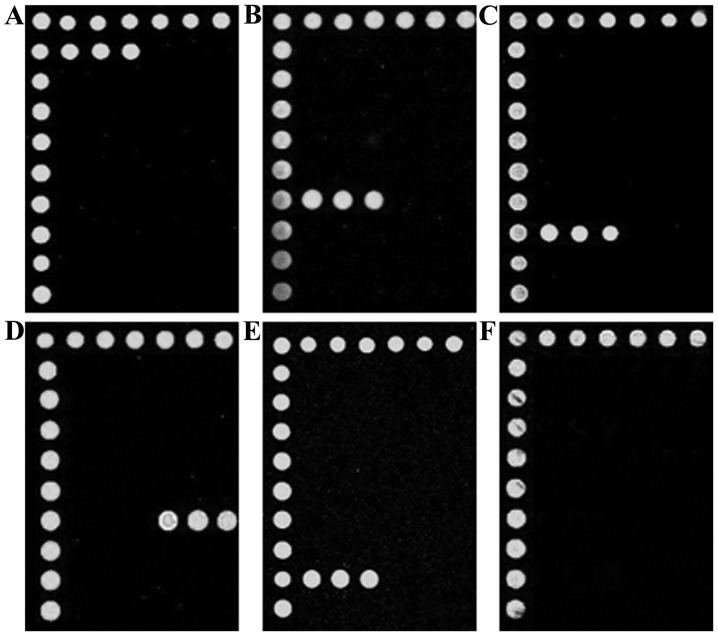
Identification of the bacterial species using microarray. (A) *Staphylococcus aureus*; (B) *Klebsiella pneumoniae*; (C) *Escherichia coli*; (D) *Streptococcus pneumoniae*; (E) 16S; (F) experimental water.

Following hybridization with the multiplex PCR products and specific probes, the microarray showed green fluorescence at the corresponding sites (Fig. 3), whereas the negative control showed no fluorescence. As a result, the bacterial species and resistance genes were identified.

### Microarray comparison with CSF culture results

**Figure 4:**
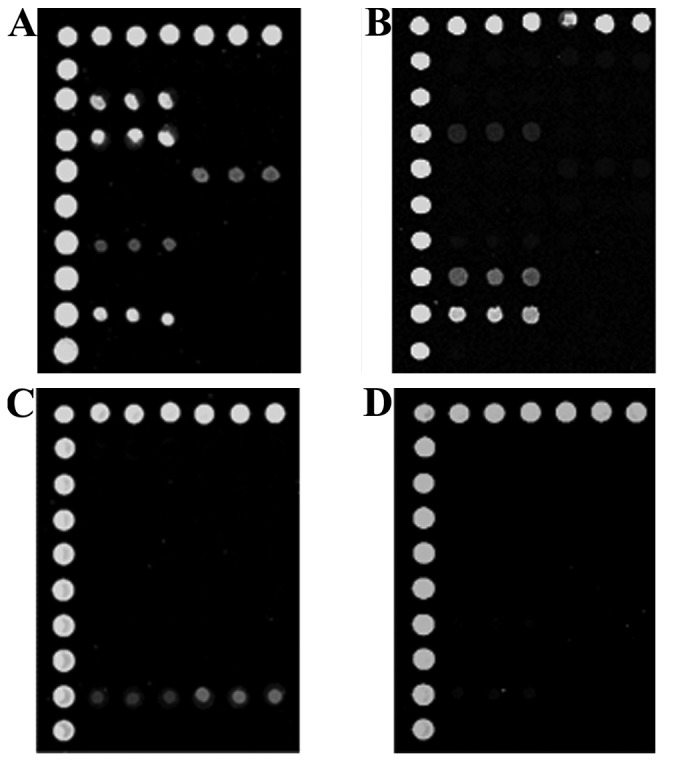
Examples of microarray detection. (A) *Klebsiella pneumoniae*, 16S, CTX-M, TEM and SHV; (B) *Escherichia coli*, 16S and TEM; (C) 16S and mecA; (D) negative.

The microarray procedure took one day, whereas bacterial culture and sensitivity testing took 4–5 days. A total of 12 CSF samples with positive bacterial cultures were identified as being positive for bacterial strains and resistance genes (Fig. 4A) using microarray, including five that were positive for *S. aureus*, three for *K. pneumonia*, two for *E. coli* and two for *S. pneumoniae*, consistent with the bacterial culture results. Among the 18 specimens that had negative bacterial culture results, bacteria and drug resistance genes were identified in a number of samples (Fig. 4B), including one sample positive for *S. aureus* and two for *E. coli*. The 16S gene without bacteria was detected in eight cases. However, the majority of these 8 cases (six) were positive for resistance genes (Fig. 4C). Samples from seven patients were without detectable 16S or drug resistance genes (Fig. 4D).

## Discussion

Although CSF bacterial culture is an important technique for the diagnosis of intracranial bacterial infection, the positive rate is too low and the process is time consuming (2,7). These disadvantages hinder the application of CSF bacterial culture in clinical treatment; therefore, various testing methods have been proposed as a supplement or substitute for bacterial culture, for example PCR (2,3,8) and immunological analyses (9). Multiplex PCR technology involves adding various specific primers in the same PCR system to simultaneously detect multiple pathogens or resistance genes (2,10). With regard to microarray technology, a large number of probes must be fixed onto a support to detect and analyze a variety of sample sequences (11). Since numerous pathogens cause intracranial infection, the two technologies were combined in the present study to detect four types of bacteria and six resistance genes. All experiments were performed in one day, showing that the process was an efficient means of genetic testing (12), facilitating the rapid detection of pathogens causing intracranial infection.

Twelve cases of positive bacterial culture specimens were identified to have the same strains based on the microarray results. Among the 18 cases of culture-negative specimens, 11 were shown to be positive following gene chip hybridization of the 16S gene, demonstrating the presence of bacterial infection. This indicated that microarray technology had a higher sensitivity than CSF culture. Eight cases were shown to be positive for the 16S gene without bacteria being identified. This may have been due to the pathogens not belonging to one of the four species included in the study design. The 15 positive specimens (12 positive in culture and 3 negative in culture but positive in microarray) showed only one result, indicating that microarray technology had a high specificity.

Among the 30 CSF samples that were diagnosed as having intracranial bacterial infections, seven cases failed to pass the gene chip detection for the presence of bacteria. There were several possible reasons for this. The experiments were conducted using a fluorescent-labeling method to perform the gene hybridization and interpret the results. When the conventional material for the fluorescent-labeling method was used, the sensitivity was low (13). In addition, the bacterial DNA content of specific samples was too low. The required level of fluorescently-labeled DNA was not achieved, even following several amplifications. Furthermore, steric hindrance existed between the target molecule and probe, and the hybridization probe molecules affected the quality of results (14). Therefore, negative microarray results were not able to be used as a reliable indicator of a definitive negative clinical diagnosis. In addition, microarray is not recommended for patients with a high positive rate of bacterial culture, such as lung infection.

Antibiotic resistance is a common phenomenon, particularly when antibiotics are frequently used (15). Microarray technology is capable of rapidly detecting common resistance genes in the case of unknown bacteria, in accordance with the various resistance genes supplying direction for early clinical trials of drugs (16). TEM, CTX-M and OXA-23 are hyperspectral β-lactamases that have been associated with drug resistance to penicillin and cephalosporins (17). mecA is a type of methicillin-resistant gene, and has been associated with drug resistance to gentamicin, imipenem and cephalosporins (18). Furthermore, the KPC gene encodes carbapenem, which has been associated with drug resistance to imipenem (19). Results from the experimental detection of resistance genes corroborated with the susceptibility test results, showing the reliability of the experimental detection of resistance genes. Of course, the detection of resistance genes also has significant limitations, for it may only help to avoid the use of partial tolerant antibiotics. However, resistance gene detection is not able to guide the selection of sensitive drugs and therefore is not able to substitute for susceptibility testing. Nevertheless, in the case of culture-negative CSF, the test result of resistance genes is the only reference index available.

The small chip experiments of the current study demonstrate that microarray technology has advantages in terms of speed and sensitivity compared with traditional CSF bacterial cultures. Based on the detection of resistance genes, microarray technology also avoids the use of antibiotics as soon as possible. However, specific disadvantages, such as high cost and low sensitivity, exist. The present trends in microarray technology have three main aspects (20,21): i) High-density probe analysis; ii) microanalysis of the test samples; and iii) microanalysis of the chip matrix area. These aspects further enhance detection sensitivity and greatly reduce the cost of testing. With the development of chip technology, microarray technology shows potential for the diagnosis of bacterial culture diseases with low positive rates, such as intracranial infection.

## Figures and Tables

**Table I:** Sequences of multiplex polymerase chain reaction primers and microarray hybridization probes.

Name of bacteria and resistance genes	Primer and probe sequence (5′-3′)	Product size, bp
*Staphylococcus aureus*	Sense primer: TAAAGCGATTGATGGTGATACG	238
	Antisense primer: AGCCAAGCCTTGACGAACTA	
	Probe: AGCGAGCATACGGCAATACTCGTTGACTGCCTCTTCGCTGT	
*Klebsiella pneumoniae*	Sense primer: GCCTTGACCGCTGGGAAAC	319
	Antisense primer: GGCGTATCCCGCAGATAAAT	
	Probe: CAACGCACTGACCATACCTACTTTGTTATTCGGGCCAAGC	
*Escherichia coli*	Sense primer: CATGCGGTTCAGCCACGGTT	471
	Antisense primer: GCGCCAGTATTCCGCACCAA	
	Probe: CGAATCAGTCTTGCTCATCGTCGCTATCTGGCTGACTGCTT	
Pneumococcal	Sense primer: CATTGTCTTAGGCGGAG	679
	Antisense primer: ATTGGTGTATTGACTGC	
	Probe: CGTTGCCGAGTTTCCATGTAGGTCTTTACCATAGTAGTTTTG	
16S	Sense primer: AGGAGGTGATCCAACCGCA	370
	Antisense primer: AACTGGAGGAAGGTGGGGAT	
	Probe: AGCTCACCATGTACGAACTGGGTGAATACGTTCCCGGGCCTTGT	
mecA	Sense primer: GGCTATCGTGTCACAATCGTTGACG	170
	Antisense primer: GGGTGGATAGCAGTACCTGAGCCA	
	Probe: CGTATCGACTGCATCAATCCAGATGGCAAAGATATTCAACTAACT	
OXA-23	Sense primer: ATGGAAGGGCGAGAAAAGG	127
	Antisense primer: TTGCATGAGATCAAGACCGATA	
	Probe: AGTGGATCTTGTACGTGGACCGCAAGTTCCTGATAGACTGGGACTGCC	
SHV	Sense primer: GCCTTGACCGCTGGGAAAC	319
	Antisense primer: GGCGTATCCCGCAGATAAAT	
	Probe: CGAATCAGTCTTGCTCATCGTGTCGCCCTGCTTGGCCCGGATAAC	
CTX-M	Sense primer: CGGGAGGCAGACTGGGTGT	381
	Antisense primer: TCGGCTCGGTACGGTCGA	
	Probe: CCTGACTGCAATAGATCCTGACGGCCATCACTTTACTGGTGCTGC	
TEM	Sense primer: GTCGCCGCATACACTATTCTCA	258
	Antisense primer: CGCTCGTCGTTTGGTATGG	
	Probe: GTCAGCGAGAACATGTGTACGCGGTTAGCTCCTTCGGTCCTCCG	
PBP1a	Sense primer: AGTATTCACTACTCAAATGC	557
	Antisense primer: GCTACAAATTGAGAGGTGTT	
	Probe: CACTGAACAGCTGACATACG GGCAGCGTAAGCAGCAGCCATCT	
